# **δ**-Aminolaevulinic acid-induced photodynamic therapy inhibits protoporphyrin IX biosynthesis and reduces subsequent treatment efficacy in vitro

**DOI:** 10.1038/sj.bjc.6690454

**Published:** 1999-06

**Authors:** S L Gibson, J J Havens, M L Nguyen, R Hilf

**Affiliations:** Department of Biochemistry and Biophysics and the UR Cancer Center, University of Rochester School of Medicine and Dentistry, University of Rochester, 601 Elmwood Avenue, Rochester NY 14642, USA

**Keywords:** δ-aminolaevulinic acid, protoporphyrin IX, porphobilinogen deaminase, photodynamic therapy

## Abstract

Recently, considerable interest has been given to photodynamic therapy of cancer using δ-aminolaevulinic acid to induce protoporphyrin IX as the cell photosensitizer. One advantage of this modality is that protoporphyrin IX is cleared from tissue within 24 h after δ-aminolaevulinic acid administration. This could allow for multiple treatment regimens because of little concern regarding the accumulation of the photosensitizer in normal tissues. However, the haem biosynthetic pathway would have to be fully functional after the first course of therapy to allow for subsequent treatments. Photosensitization of cultured R3230AC rat mammary adenocarcinoma cells with δ-aminolaevulinic acid-induced protoporphyrin IX resulted in the inhibition of porphobilinogen deaminase, an enzyme in the haem biosynthetic pathway, and a concomitant decrease in protoporphyrin IX levels. Cultured R3230AC cells exposed to 0.5 mM δ-aminolaevulinic acid for 27 h accumulated 6.07 × 10^−16^ mol of protoporphyrin IX per cell and had a porphobilinogen deaminase activity of 0.046 fmol uroporphyrin per 30 min per cell. Cells cultured under the same incubation conditions but exposed to 30 mJ cm^−2^ irradiation after a 3-h incubation with δ-aminolaevulinic acid showed a significant reduction in protoporphyrin IX, 2.28 × 10^−16^ mol per cell, and an 80% reduction in porphobilinogen deaminase activity to 0.0088 fmol uroporphyrin per 30 min per cell. Similar effects were evident in irradiated cells incubated with δ-aminolaevulinic acid immediately after, or following a 24 h interval, post-irradiation. There was little gain in efficacy from a second treatment regimen applied within 24 h of the initial treatment, probably a result of initial metabolic damage leading to reduced levels of protoporphyrin IX. These findings suggest that a correlation may exist between the δ-aminolaevulinic acid induction of porphobilinogen deaminase activity and the increase in intracellular protoporphyrin IX accumulation. © 1999 Cancer Research Campaign


*British Journal of Cancer* (1999) **80**(7), 998–1004
© 1999 Cancer Research Campaign
Article no. bjoc.1998.0454

### δ-Aminolaevulinic acid-induced photodynamic therapy inhibits protoporphyrin IX biosynthesis and reduces subsequent treatment efficacy in vitro

**SL Gibson, JJ Havens, ML Nguyen and R Hilf**

Department of Biochemistry and Biophysics and the UR Cancer Center, University of Rochester School of Medicine and Dentistry, University of Rochester, 601 Elmwood Avenue, Rochester NY 14642, USA

**Summary** Recently, considerable interest has been given to photodynamic therapy of cancer using δ-aminolaevulinic acid to induce protoporphyrin IX as the cell photosensitizer. One advantage of this modality is that protoporphyrin IX is cleared from tissue within 24 h after δ-aminolaevulinic acid administration. This could allow for multiple treatment regimens because of little concern regarding the accumulation of the photosensitizer in normal tissues. However, the haem biosynthetic pathway would have to be fully functional after the first course of therapy to allow for subsequent treatments. Photosensitization of cultured R3230AC rat mammary adenocarcinoma cells with δ-aminolaevulinic acid-induced protoporphyrin IX resulted in the inhibition of porphobilinogen deaminase, an enzyme in the haem biosynthetic pathway, and a concomitant decrease in protoporphyrin IX levels. Cultured R3230AC cells exposed to 0.5 mM δ-aminolaevulinic acid for 27 h accumulated $6.07 \times 10^{-16}$ mol of protoporphyrin IX per cell and had a porphobilinogen deaminase activity of 0.046 fmol uroporphyrin per 30 min per cell. Cells cultured under the same incubation conditions but exposed to 30 mJ $cm^{-2}$ irradiation after a 3-h incubation with δ-aminolaevulinic acid showed a significant reduction in protoporphyrin IX, $2.28 \times 10^{-16}$ mol per cell, and an 80% reduction in porphobilinogen deaminase activity to 0.0088 fmol uroporphyrin per 30 min per cell. Similar effects were evident in irradiated cells incubated with δ-aminolaevulinic acid immediately after, or following a 24 h interval, post-irradiation. There was little gain in efficacy from a second treatment regimen applied within 24 h of the initial treatment, probably a result of initial metabolic damage leading to reduced levels of protoporphyrin IX. These findings suggest that a correlation may exist between the δ-aminolaevulinic acid induction of porphobilinogen deaminase activity and the increase in intracellular protoporphyrin IX accumulation.

**Keywords:** δ-aminolaevulinic acid; protoporphyrin IX; porphobilinogen deaminase; photodynamic therapy

Photodynamic therapy (PDT) has been used successfully to control the growth of a variety of human malignancies (Rosenthal and Glatstein, 1994; van Hillegersberg et al, 1994; Fisher et al, 1995; Kriegmeir et al, 1996; Peng et al, 1997). Based on these clinical trials, PDT has been approved as a treatment for various cancers in the US, Canada, France, The Netherlands and Japan.

Traditionally, PDT consists of the systemic, topical or intratumoural administration of a photosensitizer, followed by a time interval to allow dye distribution and localization. Malignant lesions are then exposed to the light of an appropriate photosensitizer absorption wavelength. Photoactivation of the dye results in the formation of reactive oxygen species of which singlet oxygen ($^1O_2$) is reported to be the major species and the one primarily responsible for the ensuing toxicity. Photofrin®, a derivative of haematoporphyrin, is currently the photosensitizer that has received approval for PDT.

There are numerous ongoing studies aimed at development of ‘new generation’ photosensitizers, and many of these compounds show promise (Gomer, 1991; Pandey et al, 1995; Fan et al, 1997). Recently, there has been considerable interest in the formation of an endogenously produced photosensitizer, protoporphyrin IX (PPIX) (Malik and Lugaci, 1987; Kennedy and Pottier, 1992; Peng et al, 1997). Protoporphyrin IX is formed as the penultimate step of the haem biosynthetic pathway. The promise of this photosensitizer stems from reports that PPIX accumulates to a greater extent in malignant compared to normal tissue after administration of δ-aminolaevulinic acid (δ-ALA) (Dailey and Smith, 1984; van Hillegersberg et al, 1992; Malik et al, 1995). Although the basis for its concentration in tumour tissue is not known, this observation is being exploited in the use of δ-ALA in PDT.

Earlier, we demonstrated that retreatment of R3230AC rat mammary adenocarcinomas that had recurred after an initial course of PDT using Photofrin® as the photosensitizer was as effective in controlling tumour growth as the original course of therapy. Here, as an extension of those earlier studies, we have directed our efforts towards defining the effects that exogenous δ-ALA administration and subsequent photosensitization would produce on the enzymes in the haem biosynthetic pathway. The administration of δ-ALA circumvents the initial biosynthetic regulatory step, δ-ALA synthase (δ-ALA-S), which is feedback inhibited by haem. Presumably, this subversion of regulation facilitates the intracellular accumulation of PPIX after δ-ALA administration (Dailey and Smith, 1984; Batlle, 1993). It is inferred that the second slowest step in the haem biosynthetic pathway, porphobilinogen deaminase (PBGD) (Healey et al, 1981; Ades, 1990), would be the next rate-limiting step after δ-ALA-S is circumvented. In a recent report, we demonstrated a relationship between PBGD activity and the amount of PPIX accumulated, both of which increased in the

*Received 7 August 1998*
*Revised 28 October 1998*
*Accepted 27 November 1998*

*Correspondence to*: R Hilf

**Table 1** Effects of δ-ALA-induced photosensitization on cell proliferation and intracellular CellTracker™ concentration

| Day | Cell number × $10^5$ | CellTracker™ |
|---|---|---|
| 1 | 4.97 ± 0.59 | 0.22 ± 0.05 |
| 2 (no light) | 10.6 ± 1.60 | 0.10 ± 0.02 |
| 2 (30 mJ $cm^{-2}$) | 3.76 ± 0.41 | 0.23 ± 0.06 |

R3230AC cells were incubated with δ-ALA and CellTracker™ with or without subsequent light exposure (see Materials and Methods). The data on Day 1 represent baseline values for cell number and CellTracker™ fluorescence, expressed as relative fluorescence units per cell, obtained prior to the addition of δ-ALA or irradiation of cultures. The data listed for day 2 were obtained 24 h after cells were incubated for 3 h with δ-ALA and either exposed to 30 mJ $cm^{-2}$ irradiation or maintained in the dark. Each value represents the mean cell number or CellTracker™ concentration calculated from the results of at least three separate experiments performed in duplicate, ± s.e.m.

presence of exogenous δ-ALA (Gibson et al, 1998). Presently, we extend those findings by examining the effect photosensitization would have on PBGD activity and PPIX accumulation. The questions asked were: (1) are either PBGD activity or PPIX levels altered following δ-ALA-induced photosensitization?; (2) are photosensitized cells able to synthesize PPIX?; and (3) can cells be photosensitized with a second round of treatment?

Our results show that PBGD activity is inhibited by δ-ALA-induced photosensitization with a concomitant decrease in PPIX levels. Subsequent treatment of these cells with light resulted in reduced cytotoxicity, indicating that retreatment of lesions with δ-ALA-based PDT within 24 h after their initial exposure is compromised due to the inability of the cells to synthesize PPIX.

### MATERIALS AND METHODS

### Chemicals and reagents

All chemicals and reagents were purchased from Sigma Chemical Co. (St Louis, MO, USA) unless otherwise noted. Cell culture media and antibiotics were obtained from Grand Island Biological (Grand Island, NY, USA). Fetal bovine serum (FBS) was purchased from Atlanta Biologicals (Atlanta, GA, USA). δ-aminolaevulinic acid (δ-ALA) was obtained from Porphyrin Products (Logan, UT, USA) and Cell Tracker™ was purchased from Molecular Probes (Eugene, OR, USA).

### Cells and culture conditions

The R3230AC cell line was established from R3230AC rodent mammary adenocarcinomas. These tumours were maintained by transplantation into the abdominal region of 100–120 g Fischer female rats, using the sterile trochar technique described earlier (Hilf et al, 1965). Cells were cultured from tumour homogenates using the method of Hissin and Hilf (1978). Cells were maintained in passage culture on 100 mm diameter polystyrene dishes (Becton Dickinson, Franklin Lakes, NJ, USA) in 10 ml minimum essential medium (α-MEM) plus phenol red supplemented with 10% FBS, 50 units $ml^{-1}$ penicillin G, 50 μg $ml^{-1}$ streptomycin and 1.0 mg $ml^{-1}$ Fungizone® ('complete α-MEM'). Depending on the experiment, the medium was modified to fit desired conditions. In all cases, the medium contained penicillin G, streptomycin and Fungizone®.

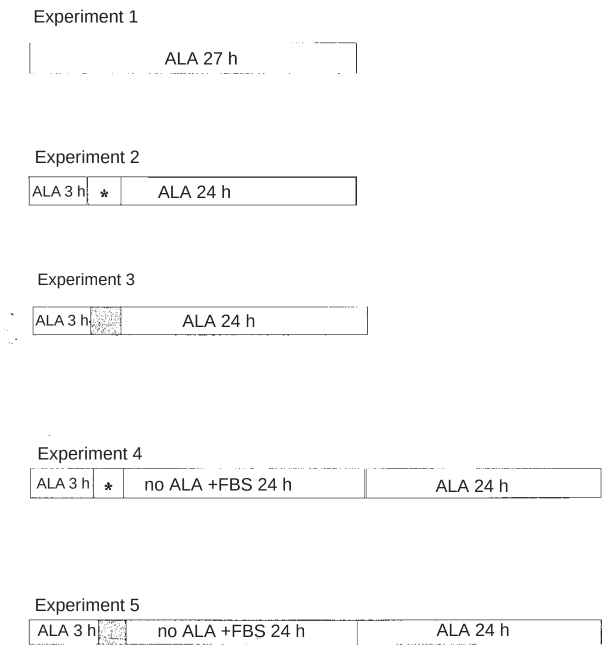

**Figure 1** Graphic representation of the experiments employed to study the effects of δ-ALA administration and subsequent photoradiation on PPIX levels and PBGD activity in cultured R3230AC cells. The horizontal boxes are not drawn to the exact time scale, but represent the different time periods in each experiment. Experiment 1: cells were incubated in α-MEM–FBS+δ-ALA in the dark for 27 h. Experiment 2: cells were incubated in α-MEM–FBS+δ-ALA for 3 h. The medium containing δ-ALA was then removed, α-MEM–FBS–phenol red–δ-ALA was added for 2.5 min (boxes with *) followed by a 24-h incubation in α-MEM–FBS+δ-ALA. Experiment 4: cells were incubated in α-MEM–FBS+δ-ALA for 3 h, and the medium replaced with α-MEM–FBS-phenol red–δ-ALA for 2.5 min (box with *). Complete α-MEM was added for 24 h, followed subsequently by a 24-h incubation in α-MEM–FBS+δ-ALA. Experiment 3 was similar to Experiment 2 except that during the 2.5-min interval between δ-ALA incubations, cultures were exposed to 30 mJ $cm^{-2}$ irradiation (shaded box). Experiment 5 was the same as Experiment 4 except that cultures were exposed to 30 mJ $cm^{-2}$ (shaded box) after the initial 3-h δ-ALA incubation period. At selected times during each experiment, cells were removed for determination of PPIX levels, PBGD activity and cell number

The modifications were made for cells incubated with δ-ALA, α-MEM without FBS containing phenol red (α-MEM–FBS+δ-ALA); or the irradiation procedure, α-MEM without FBS or phenol red or δ-ALA (α-MEM–FBS–phenol red–δ-ALA). Only cells from passages 1–10 were used for experiments. A stock of cells, from passages 1–4, were stored at –86°C and used to initiate cultures. Cultures were maintained at 37°C in a 5% carbon dioxide humidified atmosphere (Forma Scientific, Marietta, OH, USA). Passage was accomplished by trypsinizing cells and seeding new dishes with an appropriate number of cells in 10 ml of complete α-MEM. Cell counts were performed using a particle counter (Model ZM, Coulter Electronics, Hialeah, FL, USA).

### Irradiation of cultured R3230AC cells incubated with δ-ALA

Five different experimental protocols were used to determine the effects that δ-ALA incubation plus or minus light exposure had on PBGD activity, intracellular PPIX content and cell proliferation in the cultured R3230AC cells (Figure 1). In Experiment 1, cells were incubated in α-MEM–FBS+0.5 mM δ-ALA in the dark for 27 h. At

© 1999 Cancer Research Campaign selected times during this incubation period, measurements of PBGD and PPIX levels and cell counts were performed. Experiment 1 served as the baseline control for the various parameters measured, being obtained from a single 27-h δ-ALA incubation.

The second protocol, Experiment 2, consisted of incubation of cultures with α-MEM–FBS+0.5 mM δ-ALA for 3 h. The medium containing δ-ALA was removed, α-MEM–FBS–phenol red–δ-ALA was added for 2.5 min (box with asterik, Figure 1) followed by a 24-h incubation period in α-MEM–FBS+0.5 mM δ-ALA. All manipulations were performed in subdued room light, and incubations were carried out at 37°C in the dark. Experiment 2 served as a control for the cells that were exposed to δ-ALA and light, and to ascertain whether the 2.5-min interval between δ-ALA incubations would affect any of the measured parameters when compared to Experiment 1. The third protocol, Experiment 3, was similar to Experiment 2 except that the 2.5-min interval after the initial δ-ALA incubation was used to expose cultures to 30 mJ $cm^{-2}$ fluorescent light (shaded box, Figure 1). The light, emitted from a 14 W fluorescent bulb was positioned 6 cm above the monolayers, was delivered at a fluence rate of 0.2 mW $cm^{-2}$. After irradiation, cells were incubated in the dark in α-MEM–FBS+0.5 mM δ-ALA for 24 h. No interval between the end of the 2.5-min light or dark, and the addition of the second course of δ-ALA was chosen to determine the immediate effects, if any, on the measured parameters.

In the fourth protocol, Experiment 4, a 24-h dark incubation period, during which cells were maintained in complete α-MEM, was inserted between the initial 3-h δ-ALA incubation time and a subsequent 24-h δ-ALA incubation period. Finally, Experiment 5 was the same as Experiment 4, except that cultures were exposed to 30 mJ $cm^{-2}$ in α-MEM–FBS–phenol red–δ-ALA (shaded box, Figure 1). After this 24-h incubation period, α-MEM–FBS+0.5 mM δ-ALA was added for 24 additional h. The 24-h interval was added between δ-ALA incubation periods to determine if during this time, repair of photosensitized damage might occur. All determinations were made at selected times during the incubation periods in each scheme.

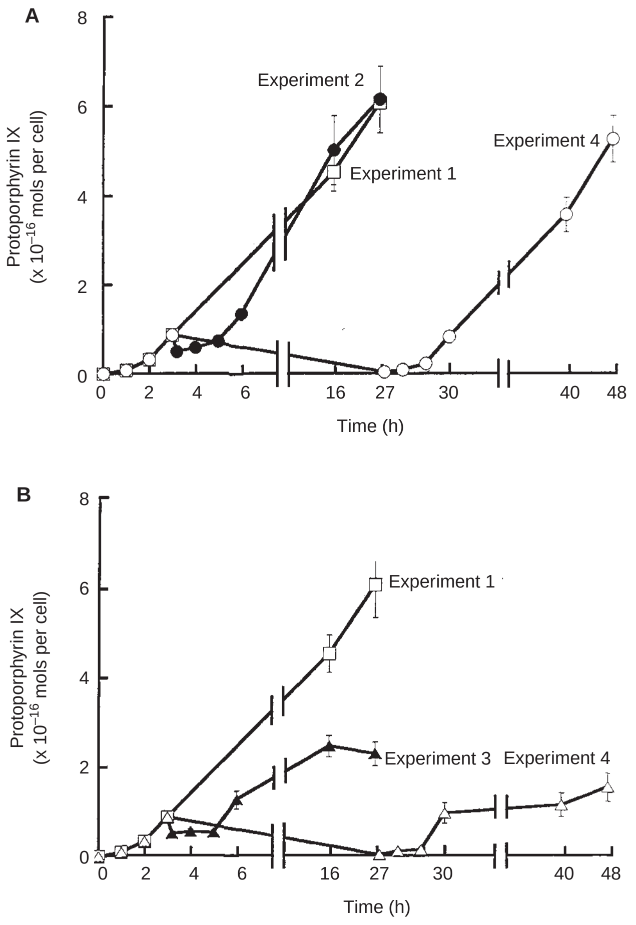

**Figure 2** Levels of intracellular PPIX measured in cultured R3230AC cells incubated over time in the presence of 0.5 mM δ-ALA. The data in Figure 2 were obtained using 5 different experimental protocols (refer to Figure 1). (**A**) The PPIX levels, expressed as mols PPIX per cell, obtained from cells exposed to Experiment 1 (□), Experiment 2 (●), or Experiment 4 (○). The data in (**B**) were obtained from experiments in which cells were exposed to either Experiment 1 (□), Experiment 3 (▲), or Experiment 5 (△) conditions. Each data point represents 4–7 separate determinations performed in duplicate, the bars are the s.e.m.

### Incubation of R3230AC cultures with CellTracker™

CellTracker™ is a commercial reagent used to fluorescently label cells as they proceed through cell division or are passed in culture. Using this reagent, one is able to track labelled cells in culture and in tissue. For our experiments, cultures were seeded and maintained as described above. The complete medium was removed, 1.0 ml of medium minus FBS plus 0.5 μM final concentration of CellTracker™ was added, and cells were incubated at 37°C for 45 min in the dark. The CellTracker™ containing medium was then removed, 1.0 ml of complete medium was added and cultures were incubated at 37°C for an additional 30 min. The complete medium was then removed and 0.5 mM δ-ALA in medium–FBS was added as described above.

### Measurement of porphyrin or CellTracker™ fluorescence in cultured cells

The extent of porphyrin biosynthesis induced by δ-ALA was determined by measuring the porphyrin fluorescence intensity in cell digests. At selected times during the incubation periods described above, the medium was removed, cells were washed once in medium minus FBS and 1.0 ml of 25% Scintigest® was added, thereby detaching cells from the surface within 5 min. The cell suspensions were transferred to 12 × 75-mm glass tubes, capped with parafilm and placed in a 37°C water bath for 1.0 h. Cell digests were then stored at –20°C until fluorescence measurements were made. Samples were removed from storage, thawed at room temperature, brought to a final volume of 2.0 ml with 1.0 ml 25% Scintigest® and mixed vigorously. Samples were then transferred to a quartz cuvette which was positioned in a spectrofluorimeter (Fluorolog 2, SPEX Industries, Edison, NJ, USA). Excitation was at 400 nm, and the fluorescence emission was scanned from 600 to 720 nm. Two distinct peaks were detected at 630 and 704 nm, with maximum fluorescence at 630 nm. The 630-nm peak was selected for measurement of intracellular porphyrin. Background autofluorescence, which represented 5% or less of the fluorescence signal at 630 nm, was determined in cells that had not been exposed to δ-ALA, and those values were subtracted from those obtained for cells exposed to δ-ALA. Intracellular porphyrin content was calculated using a reference PPIX standard dissolved

 *© 1999 Cancer Research Campaign*

in Scintigest®. Addition of the reference PPIX standard to Scintigest® containing digested cells not exposed to δ-ALA did not alter the fluorescent signal from that observed for the cell-free PPIX standard. Data are expressed as mol of fluorescent porphyrin per cell.

CellTracker™, Green CMFDA (5-chloromethylfluorescein diacetate) reagent, was incubated with cells, as described above. Once taken up into the cells, the CellTracker™ reagent is thought to undergo a glutathione-S-transferase-mediated reaction resulting in a cell-impermeant fluorescent product. Cells containing CellTracker™ were prepared for fluorescence measurements in Scintigest® as described for porphyrin determinations. Excitation of the CellTracker™/cell digest at 490 nm resulted in a single fluorescence emission peak at 540 nm and was not effected by the presence of PPIX in the cells. Intracellular fluorescence of CellTracker™ is expressed as relative fluorescence units per cell.

### Measurement of PBGD activity in cultured R3230AC cells

The activity of PBGD is measured by the absorbance of uroporphyrin, formed after light-induced oxidation of uroporphyrinogen, the immediate product of the enzymatic deamination reaction according to Grandchamp et al (1976). Briefly, cultured cells were removed with trypsin at selected times during incubations as described above, then transferred to 15-ml conical tubes and centrifuged at 1000 ***g*** for 5 min. Supernatants were discarded and 0.5 ml of distilled deionized water was added to the pellets and vigorously mixed. Cell suspensions were then sonicated using a Branson Sonicator (Model 185) at a setting of 4 for 5 s. Microscopic inspection showed that > 90% of the cells were disrupted by this procedure.

The cell lysates were centrifuged at 3000 ***g*** for 15 min and the collected supernatant was centrifuged at 10 000 ***g*** for 15 min. A portion of the supernatant containing 2 mg protein was incubated for 30 min at 45°C in the dark with 0.1 ml porphobilinogen (PBG), 1.0 mM final concentration. The reaction was stopped by addition of 2.0 ml ethyl acetate/acetic acid (3:1, v/v). The mixture was then centrifuged at 3000 ***g*** for 10 min, followed by exposure of samples to ambient light at room temperature for 15 min. Then, 1.6 ml of the porphyrin-containing upper layer were mixed with 1.0 ml of 0.5 M hydrochloric acid, followed by centrifugation at 300 ***g*** for 10 min. The supernatant was removed and fluorescence measurements were performed on the lower layer containing the uroporphyrin. One millilitre of this layer was mixed with an equivalent amount of 0.05 M phosphate-buffered saline (PBS), pH 7.1. Excitation at 405 nm resulted in a fluorescence emission peak at 650 nm. Uroporphyrin content in these samples was calculated by reference to a uroporphyrin standard dissolved in PBS. Data are expressed as fmol uroporphyrin per cell.

### Determination of cell proliferation

To determine the effect of irradiation on the proliferative capacity of cells exposed to δ-ALA, the following experiments coincident with determinations of porphyrin, PBGD and CellTracker™ levels were performed.

At selected δ-ALA incubation periods in each experiment, the medium was removed, 0.2 ml trypsin solution was added to each well and plates were incubated at 37°C until the cells lifted off the surface (approximately 5 min). Cell counts were performed using a particle counter (Model ZM, Coulter Electronics, Hialeah, FL, USA) and cell numbers from irradiated wells were compared with those from wells not exposed to light. For example, cell numbers obtained at the end of Experiment 3 were compared to values for Experiment 2.

In another series of experiments, the effects on cytotoxicity of a subsequent irradiation period in cultures previously exposed to δ-ALA and light were determined. These experiments were performed using two additional treatment regimens. In the first, after completion of all the procedures in Experiment 3, cells remaining on the plate were washed, α-MEM–FBS–phenol red–δ-ALA was added and cultures were re-exposed to 30 mJ $cm^{-2}$ light. Immediately after irradiation, complete α-MEM was added for 24 h and cell counts were obtained as above at the end of this incubation period. The same approach was used on cultures treated in Experiment 5. At the end of Experiment 5, cells were exposed to 30 mJ $cm^{-2}$, the medium was changed, complete α-MEM was added and cells were counted 24 h later as described above. The results obtained were compared to those experiments in which cells were not irradiated for a second time but had been subjected to either Experiment 2 or Experiment 4 respectively.

### Statistical analysis

Statistical analyses were performed using the Student's *t*-test. For all tests, a two-sided *P*-value of less than 0.05 was considered to be a statistically significant difference.

### RESULTS

### Effect of irradiation on δ-ALA-induced PPIX levels in vitro

The data displayed in Figure 2A demonstrate that PPIX levels in cultured R3230AC cells using Experiment 1 conditions, i.e. incubation with 0.5 mM δ-ALA for 24 h, were equivalent to that produced by cells exposed to the conditions of Experiment 2 or Experiment 4. Cells collected at the end of Experiment 1 contained $6.07 \pm 0.73 \times 10^{-16}$ mol PPIX per cell (mean ± s.e.m.), whereas cells from Experiment 2 and Experiment 4 had levels of $6.15 \pm 0.74 \times 10^{-16}$ mol and $5.26 \pm 0.53 \times 10^{-16}$ mol PPIX per cell respectively.

The data displayed in Figure 2B show that illumination of monolayer cultures using Experiment 3 or Experiment 5 regimens significantly impaired the cells' ability to synthesize PPIX after exposure to 0.5 mM δ-ALA. A 60% reduction in intracellular PPIX was observed in cultures 24 h after exposure to 30 mJ $cm^{-2}$ irradiation. The reduction in cellular PPIX content was comparable for both Experiment 3 and Experiment 5, where $2.28 \pm 0.26 \times 10^{-16}$ mol and $1.53 \pm 0.31 \times 10^{-16}$ mol of PPIX were measured respectively.

### Effect of irradiation on δ-ALA-induced PBGD activity in vitro

Concurrent experiments were performed to determine the effects of these treatment schemes on PBGD activity. Figure 3A displays data obtained from cells incubated with δ-ALA under Experiment 1 and Experiment 2, or Experiment 4 conditions. The PBGD activity after a single 24-h incubation with 0.5 mM δ-ALA (Experiment 1) was 0.046 ± 0.005 fmol uroporphyrin produced per cell. Similar PBGD activities were measured in cells from Experiment 2, 0.037 ± 0.005 fmol uroporphyrin produced per cell,

© 1999 Cancer Research Campaign

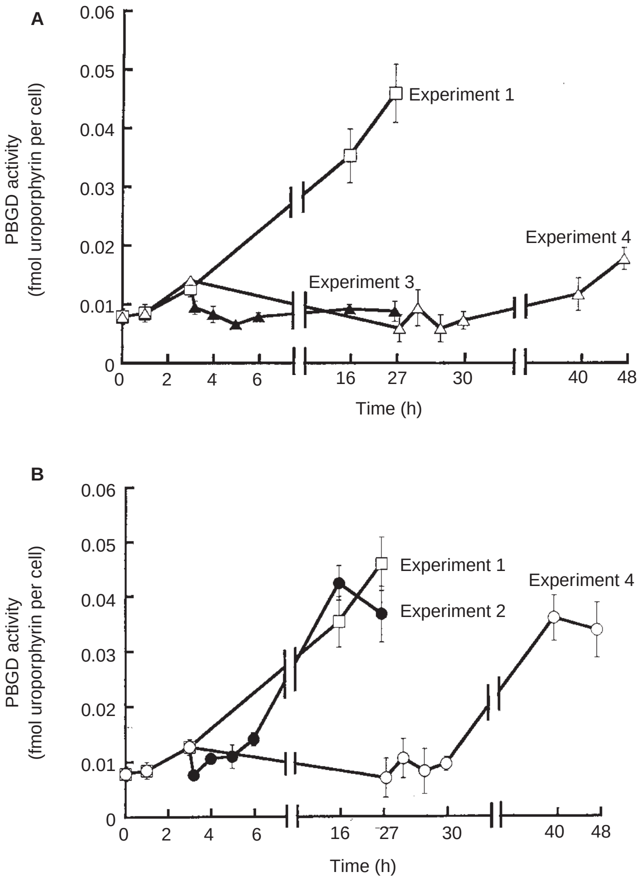

**Figure 3** Levels of PBGD activity in cultured R3230AC cells measured during incubation with 0.5 mM δ-ALA. The data in Figure 2 were obtained using five different experimental protocols (refer to Figure 1). (**A**) The PBGD levels obtained from cells exposed to Experiment 1 (□), Experiment 2 (●) or Experiment 4 (○). The data in (**B**) were obtained from experiments in which cells were exposed to either Experiment 1 (□), Experiment 3 (▲) or Experiment 5 (△) conditions. The data are expressed as fmol uroporphyrin produced per cell 30 min$^{-1}$. Each data point represents at least 4–7 separate determinations performed in duplicate, the bars are the s.e.m.

and cells from Experiment 4, 0.034 ± 0.005 fmol uroporphyrin per cell.

Exposing cell cultures to 30 mJ cm$^{-2}$ irradiation under the conditions described for Experiment 3 or Experiment 5 resulted in significant inhibition of the increase in PBGD activity observed in Experiment 1. Enzyme activity in cultures exposed to Experiment 3 was reduced to 0.0088 ± 0.0017 fmol uroporphyrin per cell and to 0.018 ± 0.0019 fmol uroporphyrin per cell in those cells treated under the Experiment 5 regimen.

### Effect of irradiation on intracellular CellTracker™ concentration and cell proliferation

Under our standard culture conditions, i.e. complete α-MEM, R3230AC cells double every 24 h (Table 1). Exposure to 0.5 mM δ-ALA for 3 h, followed immediately by 30 mJ cm$^{-2}$ irradiation, prevented the expected increase in cell number at 24 h, with cell numbers being equivalent to those at the start of the experiment. The manufacturer (Molecular Probes Inc.) states that CellTracker™ is taken up by cells equally, independent of cell cycle etc., and is distributed uniformally amongst daughter cells during cell division. The data in Table 1 demonstrate this to be the case for cultured R3230AC cells. For untreated cells that doubled in number over 24 h, concomitantly, the amount of CellTracker™ per cell was halved. However, 24 h after 3-h incubation with 0.5 mM δ-ALA and exposure to 30 mJ cm$^{-2}$ irradiation, the intracellular concentration of CellTracker™ remained the same as it was prior to photosensitization (Table 1). These data suggest that the majority of cells examined 24 h after treatment with δ-ALA and light had not proliferated.

**Table 2** Cytotoxic effects of a second application of δ-ALA-induced photosensitization on cultured R3230AC cells

| Treatment | Cell number $\times 10^5$ |
|---|---|
| 1. Control (before hv) | 3.25 ± 0.26 |
| 2. Experiment 1 | 2.48 ± 0.30 |
| 3. Experiment 2 | 1.29 ± 0.15 |
| 4. Experiment 3 | 1.12 ± 0.17 |
| 5. Experiment 4 | 0.99 ± 0.13 |
| 6. Experiment 5 | 0.68 ± 0.053 |

Cell numbers were determined 24 h after the end of each treatment (see Materials and Methods for experimental details). Treatments were: (1) cell number after 3-h incubation with 0.5 mM δ-ALA prior to 2.5 min irradiation; (2) cell number 27 h after Experiment 1; (3) cell number 24 h after Experiment 2 without a second irradiation period; (4) cell number 24 h after Experiment 3 plus a second irradiation period immediately following Experiment 3; (5) cell number after Experiment 4 without a second irradiation period; (6) cell number after Experiment 5 plus a second irradiation period following Experiment 5. Cell numbers are presented as the mean of at least three separate experiments performed in duplicate ± s.e.m.

### Cytotoxicity following a second exposure to δ-ALA-induced photosensitization

The effects that a second exposure to δ-ALA-induced photosensitization had on cell viability were determined (Table 2). Initially, exposure of cells to 0.5 mM δ-ALA for 3 h, followed by irradiation at 30 mJ cm$^{-2}$, resulted in a modest decrease in cell number ($P > 0.1$) from $3.25 \pm 0.26 \times 10^5$ cells prior to irradiation to $2.48 \pm 0.3 \times 10^5$ cells 24 h after irradiation. When 30 mJ cm$^{-2}$ irradiation was delivered to cultures at the end of the δ-ALA incubations in Experiment 3 or Experiment 5, the cell numbers declined further to $1.12 \pm 0.17 \times 10^5$ and $0.68 \pm 0.53 \times 10^5$ cells, respectively, at 24 h after irradiation. However, the decreases in cell number after the second irradiation were not significantly different from the lower cell numbers observed for Experiment 2, $1.29 \pm 0.15 \times 10^5$ cells, or Experiment 4, $0.99 \pm 0.13 \times 10^5$, when the cells were not exposed to a second irradiation cycle.

### DISCUSSION

The goal of cancer treatment is the eradication of all malignant cells. One method frequently employed is sequential treatment delivery. Multiple therapeutic courses are delivered, with the expectation that each subsequent course will destroy at least one log order of cells. Such regimens might be considered for PDT.

Previously, we examined the effectiveness of Photofrin®-based PDT on the growth of transplantable rodent mammary tumours treated at a time after their original transplantation or as tumours that recurred after an initial round of PDT (Gibson et al, 1995). We

© 1999 Cancer Research Campaign found that the second course of treatment was just as effective in controlling tumour growth as the first regimen. We also discovered that Photofrin® accumulation and subsequent phototoxicity was equivalent in cells isolated from either original or recurrent tumours. The results of those studies demonstrated that the cells surviving the first course of PDT did not develop any detectable resistance when exposed to an additional cycle of PDT. We suggested that a multiple therapeutic regimen might be employed for more than one cycle leading to enhanced treatment success. However, one major drawback to this scheme is that skin photosensitivity might be prolonged considerably if replicative treatments with Photofrin® are used.

In this report, we examine whether δ-ALA-based PDT can be applied successfully for more than one therapeutic cycle. One advantage of δ-ALA-induced PPIX production over exogenous photosensitizer administration is that PPIX does not remain in the skin for prolonged periods of time. Soon after the clearance of δ-ALA from the system, production and accumulation of PPIX rapidly decline, resulting in little latent photosensitivity. However, in contrast to Photofrin®, PDT using δ-ALA-induced photosensitization presents additional pharmacokinetic considerations. One problem is that different cell types, normal or malignant, do not respond equally to the exogenous administration of δ-ALA. This could result in less than sufficient levels of PPIX being formed in desired target tissues. Additionally, the effectiveness of a second course of PDT using δ-ALA is dependent on the presence of a fully functional haem biosynthetic pathway.

We investigated these questions, in vitro, by exposing cultured R3230AC rat mammary adenocarcinoma cells to δ-ALA and light and measuring the effects on PBGD activity, intracellular PPIX levels and cell proliferation. A second course of therapy was subsequently applied and its efficacy was assessed by determining cell proliferation. The results demonstrated that PBGD activity and PPIX levels were reduced concomitantly by the first course of δ-ALA-induced photosensitization. As expected, this limited the cells' biosynthetic capabilities to form additional PPIX, causing a significant decrease in efficacy when the second round of treatment was applied to previously treated cells. The reduced efficacy was evident for regimens with no interval between treatment cycles in Experiment 3, or with 24 h intervening between treatment courses in Experiment 5. We selected these two treatment regimens to determine whether the effects observed immediately after irradiation would persist, or if repair processes might restore haem biosynthesis. The results suggest that the damage that occurs immediately after irradiation persists for at least 24 h, as evidenced by the dramatic reduction in PPIX accumulation. The inability of cells to repair the δ-ALA-induced damage was reflected by the reduced cytotoxicity observed after a second round of irradiation was applied. Cell counts, performed 24 h after a second irradiation, were only reduced by 10–13% compared to their unirradiated counterparts. The data also show that both PBGD and PPIX levels were reduced by δ-ALA-induced photosensitization using either Experiment 3 or Experiment 5 conditions. These results lend support to the hypothesis that PBGD is a most important enzyme target when δ-ALA is administered exogenously (Gibson et al, 1998).

Our data, however, are in contrast with results obtained earlier by He et al (1993, 1995). In two separate studies they reported increased levels of PPIX in either A431 human epidermal carcinoma cells or transformed human microvascular endothelial cells at 2–48 h after irradiation of cultures in the presence of δ-ALA-induced PPIX. Ferrochelatase, the last enzyme in the haem biosynthetic pathway, catalyses the formation of haem, a non-photosensitizer, by metallation of PPIX with iron. The above reports stated that ferrochelatase activity was inhibited, attributing the increase in PPIX to a reduced ability to metallate PPIX. The difference between their data and ours might be attributed to the use of different cell types and experimental conditions. One difference is that they used confluent cultures exposed to δ-ALA and light while we performed experiments with cells in log phase growth. According to earlier reports (Washbrook et al, 1997; Wyld et al, 1997; Moan et al, 1998) and our unpublished results, δ-ALA uptake, PPIX accumulation and PBGD activity are dependent on cell type and cell density. This latter phenomenon could be a major confounding factor in comparisons of data obtained by different groups.

On the other hand, van der Veen et al (1994) reported that a transplantable tumour treated with δ-ALA-based PDT displayed PPIX levels that were reduced to below background immediately after irradiation. Ninety minutes later, PPIX fluorescence reappeared in the tumours at half the level observed prior to the initial light exposure. They attributed these events to PPIX photobleaching during the first light exposure followed by resynthesis of PPIX prior to the second irradiation cycle. Those data are similar to ours, but their attribution that disappearance of PPIX fluorescence is entirely due to photobleaching is speculative. They reported that their first course of therapy altered the structural integrity of the tumours, results suggesting that the damaged cells might release PPIX into the extracellular space where it could be transported from the tumour site. This occurrence by itself could contribute to the apparent loss in porphyrin fluorescence. In our experiments in vitro, we did not detect a reduction in porphyrin fluorescence immediately after irradiation, essentially ruling out photobleaching. Thus, the reduced fluorescence we see 24 h after irradiation is likely due to the inability of cells to synthesize PPIX, a hypothesis supported by the data showing inhibition of PBGD activity.

The results obtained here, taken together with earlier reports, demonstrate that δ-ALA-induced porphyrin biosynthesis, and the effect that irradiation has on this process, is quite complicated. Our data demonstrate that one component of the haem biosynthetic pathway, PBGD, is sensitive to PPIX photosensitization. We also show an apparent association between inhibition of this enzyme and reduction in PPIX synthesis. Finally, these results suggest that if lesions are re-treated with δ-ALA-induced PDT within the first 24 h after initial therapy, the subsequent treatment efficacy would be compromised by the diminished ability of cells to synthesize PPIX. We plan to examine this hypothsis in vivo and to continue to measure the haem biosynthetic pathway to determine whether any other key control points are affected by δ-ALA-induced photosensitization.

### ACKNOWLEDGEMENTS

We acknowledge the assistance of Debbie Pilc of the Animal Tumor Research Facility of the University of Rochester Cancer Center (CA11198) for the transplantation and maintenance of rodent tumours. This research was supported by Grant CA36856 from the NCI, National Institutes of Health, USA.

### REFERENCES

Ades IZ (1990) Haem production in animal tissues: the regulation of biogenesis of δ-aminolevulinate synthase. *Int J Biochem* **22**: 565–578

Batlle AM del C (1993) Porphyrins, porphyrias, cancer and photodynamic therapy – a model for carcinogenesis. *J Photochem Photobiol B: Biol* **20**: 5–22

© 1999 Cancer Research Campaign 

Dailey HA and Smith A (1984) Differential interaction of porphyrins used in photoradiation therapy with ferrochelatase. *Biochem J* **223**: 441–445.

Fan KFM, Hopper C, Speight PM, Buonaccorsi GA and Bown SG (1997) Photodynamic therapy using mTHPC for malignant disease in the oral cavity. *Int J Cancer* **73**: 25–32

Fisher AM, Murphee AL and Gomer CJ (1995) Clinical and preclinical photodynamic therapy. *Lasers Surg Med* **17**: 2–31.

Gibson SL, Nguyen ML, Foster TH, White G and Hilf R (1995) Efficacy of photodynamic therapy on original and recurrent rat mammary tumors. *Photochem Photobiol* **61**: 196–199.

Gibson SL, Cupriks DJ, Havens JJ, Nguyen ML and Hilf R (1998) A regulatory role for porphobilinogen deaminase (PBGD) in δ-aminolaevulinic acid (δ-ALA)-induced photosensitization? *Br J Cancer* **77**: 235–243.

Gomer CJ (1991) Preclinical examination of first and second generation photosensitizers used in photodynamic therapy. *Photochem Photobiol* **54**: 1093–1107

Grandchamp B, Phung N, Grelier M and Nordmann Y (1976) The spectrophotometric determination of uroporphyrinogen I synthetase activity. *Clin Chem Acta* **70**: 113–118.

He D, Sassa S and Lim HW (1993) Effect of UVA and blue light on porphyrin biosynthesis in epidermal cells. *Photochem Photobiol* **57**: 825–829

He D, Behar S, Nonaura N, Sassa S and Lim HW (1995) The effect of ALA and radiation on porphyrin/haem biosynthesis in endothelial cells. *Photochem Photobiol* **61**: 656–661

Healey JF, Bonkowsky HL, Sinclair PR and Sinclair JF (1981) Conversion of 5-aminolaevulinate into haem by liver homogenates; comparison of rat and chick embryo. *Biochem J* **198**: 595–604

Hilf R, Michel I, Bell C, Freeman JJ and Borman A (1965) Biochemical and morphological properties of a new lactating tumor line in the rat. *Cancer Res* **25**: 286–299

Hissin PJ and Hilf R (1978) Effect of insulin in vivo and in vitro on amino acid transport into cells from R3230AC mammary adenocarcinoma and their relationship to tumor growth. *Cancer Res* **38**: 3646–3651

Kennedy JC and Pottier RH (1992) Endogenous protoporphyrin IX, a clinically useful photosensitizer for photodynamic therapy. *J Photochem Photobiol* **14**: 275–299

Kriegmeir M, Baumgartner R, Kneuchel R, Stepp H, Hofsteder F and Hofstetter A (1996) Detection of early bladder cancer by 5-aminolevulinic acid induced porphyrin fluorescence. *J Urol* **155**: 105–110

Malik Z and Lugaci H (1987) Destruction of erythroleukaemic cells by photoactivation of endogenous porphyrins. *Br J Cancer* **56**: 589–595

Malik Z, Kostenich G, Roitman L, Ehrenberg B and Orenstein A (1995) Topical application of 5-aminolevulinic acid, DMSO and EDTA: protoporphyrin IX accumulation in skin and tumours of mice. *J Photochem Photobiol B: Biol* **28**: 213–218

Moan J, Bech O, Gaullier J-M, Stokke T, Steen HB, Ma LW and Berg K (1998) Protoporphyrin IX accumulation in cells treated with 5-aminolevulinic acid: dependence on cell density, cell size and cell cycle. *Int J Cancer* **75**: 134–139

Pandey RK, Potter WR, Meunier I, Sumlin AB and Smith K (1995) Evaluation of new benzoporphyrin derivatives with enhanced PDT efficacy. *Photochem Photobiol* **62**: 764–768

Peng Q, Berg K, Moan J, Kongshaug M and Nesland JM (1997) 5-aminolevulinic acid-based photodynamic therapy: principles and experimental research. *Photochem Photobiol* **65**: 235–251

Rosenthal DI and Glatstein E (1994) Clinical applications of photodynamic therapy. *Ann Med* **26**: 405–409

Van der Veen N, van Leengoed HLLM and Star WM (1994) In vivo fluorescence kinetics and photodynamic therapy using 5-aminolevulinic acid-induced porphyrin: increased damage after multiple irradiations. *Br J Cancer* **70**: 867–872

van Hillegersberg R, Van den Berg JW, Kort WJ, Terpstra OT and Wilson JH (1992) Selective accumulation of endogenously produced porphyrin in a liver metastasis model in rats. *Gastroenterology* **103**: 647–651

van Hillegersberg R, Kort WJ and Wilson JH (1994) Current status of photodynamic therapy in oncology. *Drugs* **48**: 510–527

Washbrook R, Fukuda H, Batlle A and Riley P (1997) Stimulation of tetrapyrrole synthesis in mammalian epithelial cells in culture by exposure to aminolaevulinic acid. *Br J Cancer* **75**: 381–387

Wyld L, Burn JL, Reed AWR and Brown NJ (1997) Factors effecting aminolevulinic acid-induced generation of protoporphyrin IX. *Br J Cancer* **76**: 705–712

*© 1999 Cancer Research Campaign*
